# Treadmill Running Ameliorates Destruction of Articular Cartilage and Subchondral Bone, Not Only Synovitis, in a Rheumatoid Arthritis Rat Model

**DOI:** 10.3390/ijms19061653

**Published:** 2018-06-03

**Authors:** Seiji Shimomura, Hiroaki Inoue, Yuji Arai, Shuji Nakagawa, Yuta Fujii, Tsunao Kishida, Shohei Ichimaru, Shinji Tsuchida, Toshiharu Shirai, Kazuya Ikoma, Osam Mazda, Toshikazu Kubo

**Affiliations:** 1 Department of Orthopaedics, Graduate School of Medical Science, Kyoto Prefectural University of Medicine, Kawaramachi-Hirokoji, Kamigyo-ku, Kyoto 602-8566, Japan; s-shimo@koto.kpu-m.ac.jp (S.S.); hinoue@koto.kpu-m.ac.jp (H.I.); y-fujii@koto.kpu-m.ac.jp (Y.F.); katsura@koto.kpu-m.ac.jp (S.I.); tuchi-kf@koto.kpu-m.ac.jp (S.T.); shirai.t77@gmail.com (T.S.); kazuya@koto.kpu-m.ac.jp (K.I.); orthoped@koto.kpu-m.ac.jp (T.K.); 2 Department of Sports and Para-Sports Medicine, Graduate School of Medical Science, Kyoto Prefectural University of Medicine, Kawaramachi-Hirokoji, Kamigyo-ku, Kyoto 602-8566, Japan; shushi@koto.kpu-m.ac.jp; 3 Department of Immunology, Graduate School of Medical Science, Kyoto Prefectural University of Medicine, Kawaramachi-Hirokoji, Kamigyo-ku, Kyoto 602-8566, Japan; tsunao@koto.kpu-m.ac.jp (T.K.); mazda@koto.kpu-m.ac.jp (O.M.)

**Keywords:** treadmill running, exercise, articular cartilage, rheumatoid arthritis, collagen-induced arthritis, pro-inflammatory cytokine, cnnexin43, osteoporosis

## Abstract

We analyzed the influence of treadmill running on rheumatoid arthritis (RA) joints using a collagen-induced arthritis (CIA) rat model. Eight-week-old male Dark Agouti rats were randomly divided into four groups: The control group, treadmill group (30 min/day for 4 weeks from 10-weeks-old), CIA group (induced CIA at 8-weeks-old), and CIA + treadmill group. Destruction of the ankle joint was evaluated by histological analyses. Morphological changes of subchondral bone were analyzed by μ-CT. CIA treatment-induced synovial membrane invasion, articular cartilage destruction, and bone erosion. Treadmill running improved these changes. The synovial membrane in CIA rats produced a large amount of tumor necrosis factor-α and Connexin 43; production was significantly suppressed by treadmill running. On μ-CT of the talus, bone volume fraction (BV/TV) was significantly decreased in the CIA group. Marrow star volume (MSV), an index of bone loss, was significantly increased. These changes were significantly improved by treadmill running. Bone destruction in the talus was significantly increased with CIA and was suppressed by treadmill running. On tartrate-resistant acid phosphate and alkaline phosphatase (TRAP/ALP) staining, the number of osteoclasts around the pannus was decreased by treadmill running. These findings indicate that treadmill running in CIA rats inhibited synovial hyperplasia and joint destruction.

## 1. Introduction

Rheumatoid arthritis (RA) is an autoimmune disease in which the synovium is the primary target tissue. Pro-inflammatory cytokines, such as tumor necrosis factor (TNF)-α, interleukin (IL)-6, IL-1b, and stromal cell-derived factor 1 (CXCL 12), are produced excessively in the synovium of patients with RA, resulting in joint destruction, joint swelling, arthralgia, limited range of motion, and other complications [1,2,3]. RA-related systemic complications affect major important organs including the heart, lungs, liver, and/or brain [4]. The progression of these local and global symptoms reduce activities of daily life (ADL) and lead to various complications.

Pharmacotherapy is the basic treatment for RA. The advent of biological disease-modifying anti-rheumatic drugs (bDMARDs) caused a paradigm shift in RA treatment and made possible the minimization of joint destruction [5,6,7]. However, prescription of bDMARDs is limited because of their adverse effects and/or cost [8,9]. Therefore, the development of additional drug therapies is necessary.

Exercise therapy is useful not only for orthopedic diseases but also for various systemic diseases because of its safety and convenience. Exercise therapy includes stretching, muscle training, range of motion training, and others. Among them, exercise therapy using the treadmill has protective effects on the articular cartilage and subchondral bones in patients with osteoarthritis (OA); in healthy individuals [10] it suppresses osteophyte formation, bone destruction [11], and it also improves joint symptoms associated with OA. It was reported that treadmill exercise contributes to the improvement of cardiopulmonary function and improvement in ADL in patients with cardiac and/or pulmonary diseases [12,13]. Thus, exercise therapy contributes to improvement, not only of joint symptoms, but also of systematic improvement in ADL. It has been reported that exercise therapy for RA also ameliorates joint symptoms [14] and improves physical function and ADL, with improvement of muscle strength and cardiopulmonary function [15]. A Cochrane review strongly recommends systemic exercise therapy for RA [16], but the molecular and biological mechanisms of exercise therapy for RA have not been elucidated, and the effects on joint destruction remain unknown. An analysis of the influence of exercise therapy on joints in RA could lead to the development of appropriate exercise therapy for RA. Based on this evidence, the objective of this study was to evaluate the influence of exercise therapy for active RA on articular cartilage and subchondral bone using a collagen-induced arthritis (CIA) animal model.

## 2. Results

### 2.1. The Effect of Treadmill Running on Articular Cartilage

**Figure 1:**
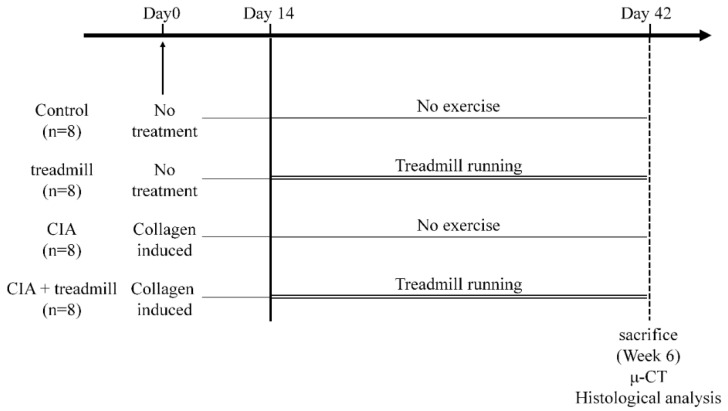
Experimental protocols. Eight-week-old male Dark Agouti rats were randomly divided into four groups: Control group, treadmill group (12 m/min, 30 min/day for 4 weeks from 10-week-old), collagen-induced arthritis (CIA) group (induced CIA at 8-week-old), and CIA + treadmill group. Histological and immunohistochemical analyses were performed to evaluate the degeneration of articular cartilage, synovitis in the ankle joint, and erosion in subchondral bone. Morphological changes of subchondral bone were analyzed by μ-CT.

**Figure 2:**
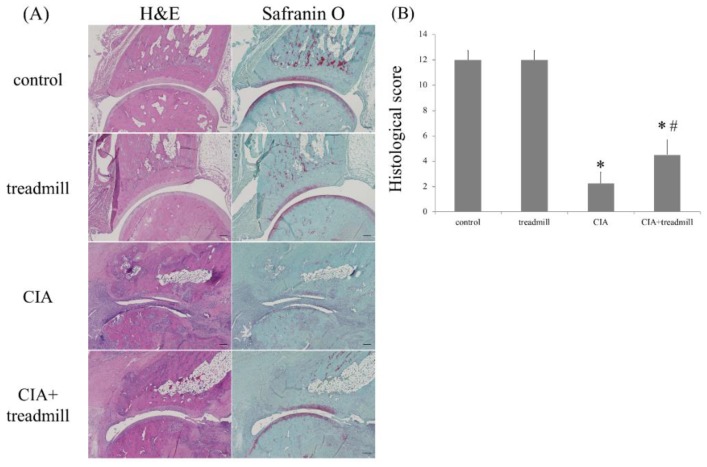
Treadmill running in the collagen-induced arthritis (CIA) rat model suppressed destruction of the ankle joint. On day 42, the ankle joints were sectioned. Representative microscopic images of hematoxylin and eosin and safranin O-stained sagittal sections (**A**), as well as the histological scores (means ± standard deviation) (**B**), are shown. * *p* < 0.01 versus control group, # *p* < 0.01 versus CIA group. *p*-value was calculated using the Tukey–Kramer test as post–hoc analysis of the analysis of variance. Scale bar = 200 μm.

To evaluate the effects of treadmill running on articular cartilage in CIA rats, the right paw was evaluated by histological examination with hematoxylin and eosin (H & E) or safranin O staining on day 42 of our protocol (Figure 1 and Figure 2A).

Histological examination of the paws of the rats in the CIA group 42 days post-immunization showed typical signs of RA, including prominent hyperplasia of the synovium, massive infiltration of inflammatory cells into the articular cavity, degeneration of the articular cartilage caused by pannus, and bone erosion. In contrast, histological examination of the paws of the CIA + treadmill group of rats at 42 days post-immunization and after 28 days of treadmill running, showed that inflammatory cell infiltration and synovial membrane proliferation were significantly suppressed compared to those in the CIA group and that destruction of joint structure was also significantly suppressed compared to that in the CIA group. The stainability of articular cartilage of the ankles stained with safranin O in the CIA + treadmill group of rats was also improved compared to those in the CIA group (Figure 2A). Histological arthritis scoring also indicated that the manifestations of RA were significantly milder in CIA + treadmill group of rats than in CIA group of rats (Figure 2B).

### 2.2. Influence of Treadmill Running on TNF-α and Cx43 in Synovium

**Figure 3:**
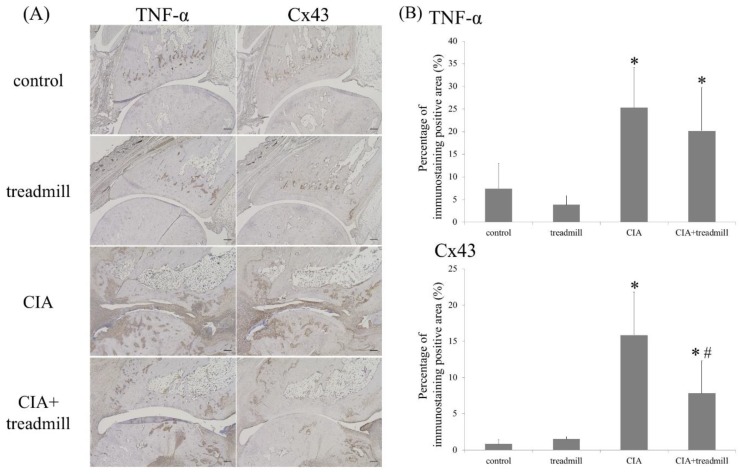
Treadmill running in collagen-induced arthritis (CIA) rat model suppressed TNF-α and Cx43. On day 42, the ankle joints were sectioned. Representative microscope images of TNF-α and Cx43 immunohistochemical staining are shown (**A**) and all images were evaluated semi-quantitatively using ImageJ (**B**). * *p* < 0.01 versus control group, # *p* < 0.01 versus CIA group. *p*-value was calculated using the Tukey–Kramer test as post–hoc analysis of the analysis of variance. Scale bar = 200 μm.

We evaluated the effects of treadmill running on pro-inflammatory cytokines in the synovium using ImageJ (National Institutes of Health, Bethesda, MD, USA) (Figure 3). TNF-α was evaluated by immunohistochemistry as a representative pro-inflammatory cytokine. Cx43 was also evaluated because we consider that Cx43 may be a key gene associated with RA onset [17]. Production of TNF-α in the synovial membrane increased in the CIA group; this increase tended to be suppressed in the CIA + treadmill group. In addition, production of Cx43 increased in the CIA group; this increase was also suppressed in the CIA treadmill group. The increase in Cx43 production was similar to that of TNF-α.

### 2.3. Prevention of Bone Loss in RA Model by Treadmill Running

**Figure 4:**
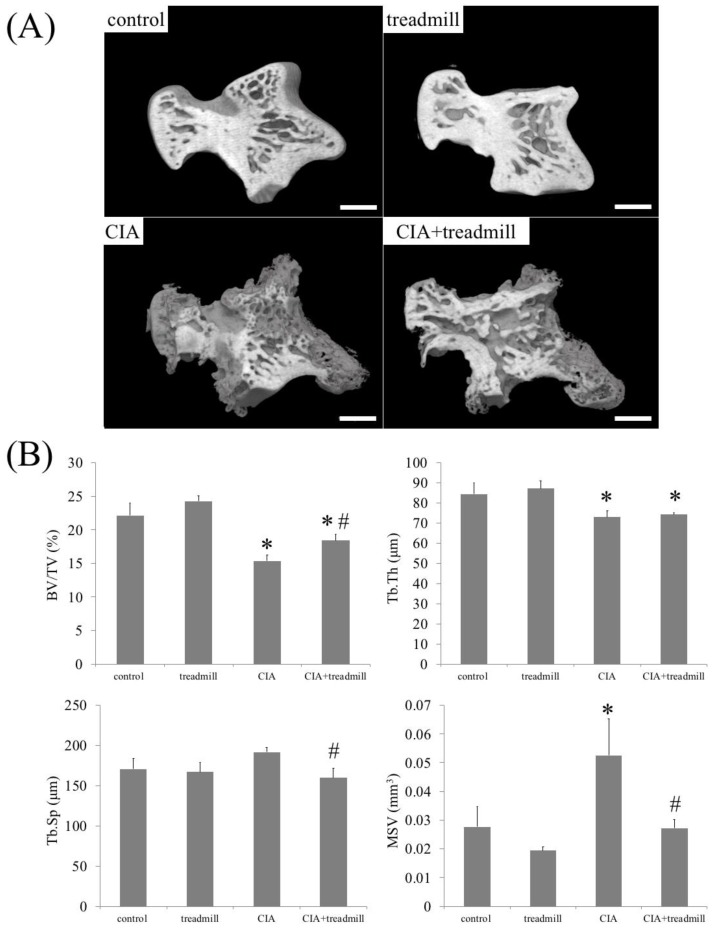
Treadmill running in collagen-induced arthritis (CIA) rat model improves bone loss. On day 42, morphological changes of cancellous bone were analyzed by μ-CT. Representative three-dimensional reconstruction of the sagittal sections of talus architecture (**A**) and trabecular bone parameters in the talus such as trabecular bone volume fraction (BV/TV), trabecular thickness (Tb.Th), trabecular spacing (Tb.Sp), and marrow star volume (MSV) of the whole talus (**B**) are shown. * *p* < 0.01 versus control group, # *p* < 0.01 versus CIA group. Scale bar = 1 mm.

We investigated whether bone composition around an inflamed joint changed with treadmill running using μ-CT (Figure 4). μ-CT analyses revealed that CIA reduced the trabecular bone volume fraction (BV/TV) and trabecular bone thickness (Tb.Th) in the talus, and treadmill running increased BV/TV in CIA rats. In CIA rats, forced running for 4 weeks partially improved trabecular spacing (Tb.Sp) and marrow star volume (MSV) compared to the control group level.

### 2.4. The Effects of Treadmill Running on Bone Erosion and Formation in CIA Rats

**Figure 5:**
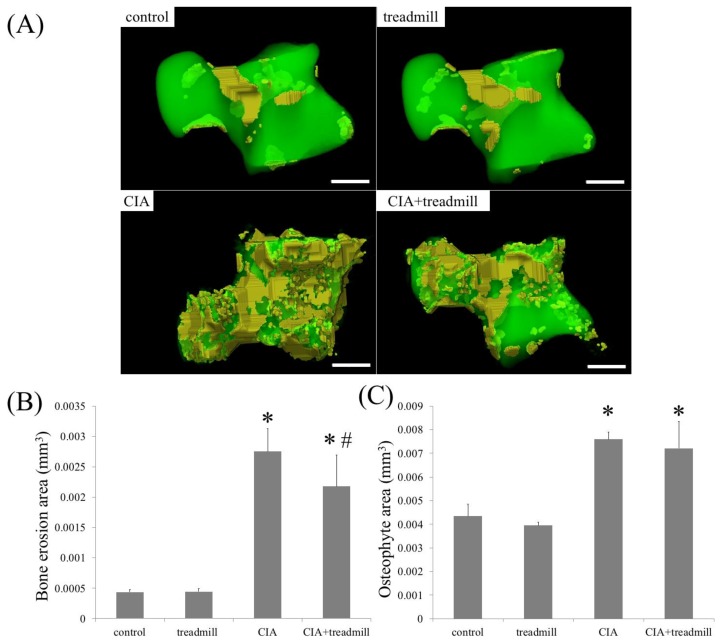
Treadmill running in collagen-induced arthritis (CIA) rat model suppressed bone erosion. On day 42, volume of bone erosion and osteophyte of whole talus were analyzed by μ-CT. Representative 3D reconstruction of bone erosion area (**A**) in the whole talus architecture. Yellow area is bone erosion area. The volume of the bone erosion area (**B**) and osteophyte area (**C**) calculated using 3D-micro-CT were shown. * *p* < 0.01 compared to the control group rats, # *p* < 0.01 compared to the CIA group rats. Scale bar = 1 mm.

We investigated whether treadmill running affected the amount of bone erosion and osteoblast activity (Figure 5). μ-CT analyses revealed that CIA induced bone erosion in the talus, and treadmill running reduced this amount in CIA rats. In CIA rats, the amount of osteophyte area did not change regardless of the presence or absence of treadmill running.

**Figure 6:**
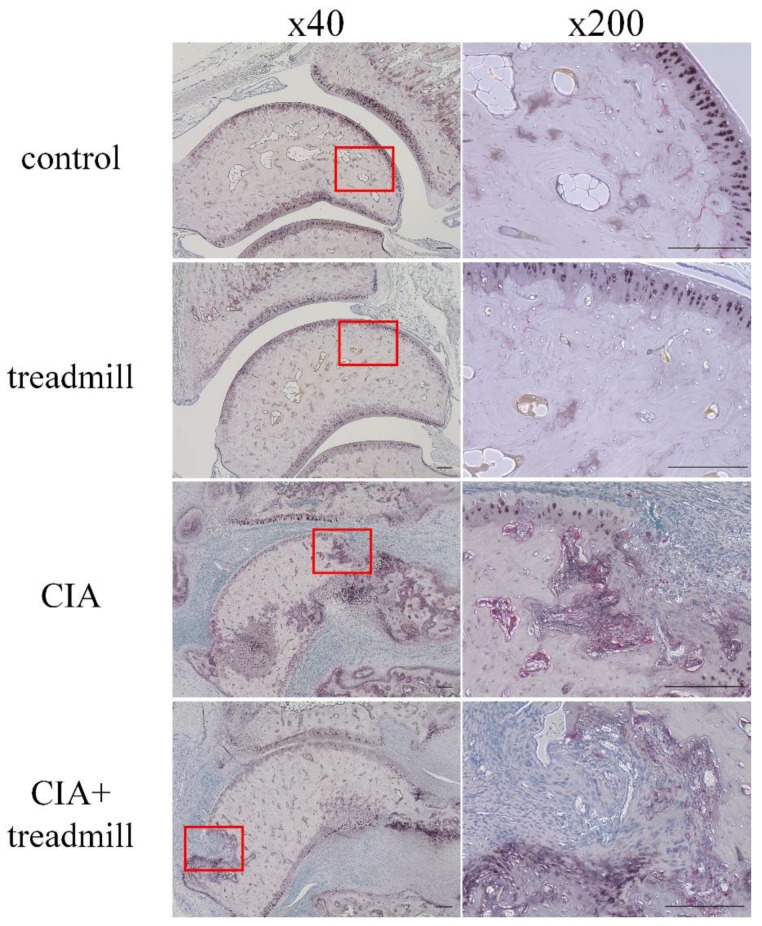
Treadmill running in collagen-induced arthritis (CIA) rat model suppressed differentiation of osteoclasts and accelerated osteoblast activity. On day 42, the ankle joints were sectioned. Representative microscopic images of tartrate-resistant acid phosphate (TRAP) and alkaline phosphatase (ALP)-stained sagittal sections are shown. Pannus formation within the red frame was examined, and magnified images were shown on the right panels. Cells stained red are osteoclasts; area stained brown is ALP positive. Scale bar = 100 μm.

We evaluated the effects of treadmill running on bone metabolism using tartrate-resistant acid phosphate and alkaline phosphatase (TRAP/ALP) staining (Figure 6). The number of TRAP-positive cells was significantly lower in the areas of pannus invasion into the ankle joints of the CIA + treadmill group than in the CIA group. In contrast, the volume of the ALP-positive area was significantly larger in areas of pannus invasion into the ankle joints of the CIA + treadmill group than in the CIA group.

## 3. Discussion

Treadmill running is a typical exercise therapy and applicable not only for health promotion but also for prevention and treatment of OA. Appropriate treadmill running suppresses joint destruction and improves symptoms by strengthening periarticular muscles and providing moderate mechanical stress on articular cartilage and subchondral bone. However, higher intensity treadmill running leads to adverse effects on the joints. Although the appropriate running load remains unknown, OA progression was suppressed by treadmill running at a speed of 12 m/min in a rat OA model [18]. On the other hand, OA was induced in normal rats with treadmill running at 26.8 m/min [19]. Thus, we selected 12 m/min treadmill running, which was demonstrated to have joint-protective effects in the rat OA model, although the joint condition is different between RA and OA. As a result, we showed that 12 m/min treadmill running suppressed inflammation and hyperplasia of the synovium and the destruction of articular cartilage and subchondral bone of the ankles; additionally, the histological arthritis score was improved in CIA rats. Thus, we found that running at 12 m/min is an exercise intensity that can improve arthritis in RA. We revealed that low-intensity treadmill running may be safe in patients with RA and may represent a form of exercise therapy capable of improving synovitis.

The main lesion of RA involves the synovial membrane; immune cells in the RA synovium produce pro-inflammatory cytokines. These cytokines increase the expression of the receptor activator of nuclear factor κ-B ligand (RANKL), which induce osteoclast differentiation, and matrix metalloproteinases (MMPs), causing joint destruction. Therefore, controlling synovitis and suppressing the production of pro-inflammatory cytokines are important for the treatment of RA and prevention of joint destruction. We focused on *Cx43* as it is a key gene associated with synovitis in RA. Connexins constitute gap junctions that allow the exchange of ions, second messengers, and metabolites between adjacent cells, and are involved in differentiation, proliferation, and inflammatory reactions. Among connexins, Cx43 is most strongly expressed in the synovial membrane. We previously reported that Cx43 in the CIA rat synovial membrane induced joint destruction via pro-inflammatory cytokines such as TNF-α and IL-6 and joint destruction was suppressed by downregulating Cx43 [17]. We also reported the cross-talk between *Cx43* and *TNF-α* in human synovial fibroblasts [20]. In the present study, Cx43 in the CIA group was produced in the synovial membrane at the same level as TNF-α. Moreover, production of Cx43 and TNF-α in the synovial membrane was suppressed in the CIA + treadmill group compared to the CIA group, likely resulting in decreased articular cartilage and bone destruction. Based on these results, we suspected that decreases in TNF-α and Cx43 may be involved in the control of arthritis by treadmill running.

Mechanical stress has been reported to be involved in the control of synovitis. Estell reported that fibroblast-like synovial cells on the synovial surface express IL-1α in response to shear stress [21]. Zhang reported that the expression of MMPs and lysyl oxidases (LOX) in synovial fibroblasts varied depending on the intensity of the applied mechanical stress [22]. In addition, King reported that synovitis in a horse OA model was suppressed by treadmill running under water, and joint symptoms were also improved [23]. From these reports, we suspected that the synovial tissue may change the intraarticular environment in response to various mechanical stresses induced by treadmill running. Meanwhile, it is known that *Cx43* in cardiomyocytes and osteocytes react to mechanical stress, such as elongation stress [24,25]; it was also reported that the absence of *Cx43* selectively from osteocytes enhances the responsiveness to mechanical force and mineralization [26]. Thus, *Cx43* changes its expression in response to mechanical stress. In the present study, we suspected that Cx43 and TNF-α produced from the synovium may be suppressed by the mechanical stress of treadmill running.

Mechanical stress is also directly applied to articular cartilage and bone during treadmill running [27]. We previously reported that moderate hydrostatic pressure on chondrocytes enhances production of the cartilage matrix, and excessive hydrostatic pressure promotes the expression of proinflammatory cytokines such as IL-6, TNF-α, and a cartilage matrix-degrading enzyme [28,29]. Therefore, we suspected that treadmill running may act as not only a suppressor of pro-inflammatory cytokine production from the synovial membrane, but also a joint protector through mechanical stress applied to the articular cartilage per se, resulting in suppression of joint destruction.

It is generally known that bone mineralization progresses through activation of osteoblasts by applying mechanical stress [30]. Therefore, low physical activity in patients with RA decreased bone formation. In this study, treadmill running in CIA rats increased the ALP positive area and suppressed subchondral bone erosion and progression of bone loss. We considered that, in the CIA rat model, mechanical stress was applied directly to bone by treadmill running, osteoblasts were activated, and bone formation was possibly promoted.

It is also known that bone erosion is dependent on the RANKL production, a TNF family member that is essential for osteoclast formation and activity, in normal and pathological states of bone remodeling. The catabolic effects of RANKL are regulated by osteoprotegerin, a TNF receptor family member that binds RANKL [31]. Recent studies reported that pro-inflammatory cytokines are important for differentiation of osteoclasts, in addition to this mechanism, and promote bone erosion [32]. In this study, treadmill running in CIA rats decreased pro-inflammatory cytokines and the number of TRAP positive cells, resulting in suppression of bone loss. Production of TNF-α and Cx43 in synovitis in CIA rats may be inhibited by treadmill running. We suspected that differentiation of osteoclasts was controlled, suppressing erosion of subchondral bone and slowing progression of bone loss.

Based on the above, we considered that treadmill running may suppress subchondral bone erosion and progression of bone loss not only through mechanical stress but also through suppression of pro-inflammatory cytokine production. We expect that patients with RA may be protected from bone erosion and osteoporosis by moderate treadmill running.

This study has several limitations. We did not evaluate TRAP/ALP staining semi-quantitatively. We tried to evaluate TRAP/ALP staining using ImageJ software, but it was difficult to differentiate TRAP-positive cells and ALP-positive cells because of their similar color range. We applied an exercise intensity that is considered appropriate for normal and rat OA models, but we did not consider whether this intensity is most appropriate for RA. We did not carry out experiments attempting to block *TNF-α* and *Cx43* expression or activity. Therefore, we cannot discuss how TNF-α and Cx43 are involved in the suppression of arthritis by treadmill running in CIA rats. In clinical practice, drug therapy such as methotrexate is started at the initial diagnosis of RA; thus, we cannot ignore the effect of drugs on synovitis inhibition. In this study, we did not consider the effects of exercise therapy under drug administration.

## 4. Materials and Methods

This study was conducted in accordance with the animal research guidelines of the Kyoto Prefectural University of Medicine, Kyoto, Japan (Code no. M25-29, 1 April 2017).

### 4.1. CIA Model

The CIA rat model is widely used for RA-related in vivo studies because of its similarity with RA in human. To induce CIA, collagen type II (Collagen Research Center, Tokyo, Japan) was dissolved in 0.01 M acetic acid (2 mg/mL) and emulsified at 1:1 in Freund’s incomplete adjuvant (CII/FIA; Sigma-Aldrich, St. Louis, MO, USA) on ice. Eight-week-old male Dark Agouti (DA) rats (Shimizu Laboratory Suppliers, Kyoto, Japan) weighing 180–230 g were intradermally injected with 200 mL CII/FIA solution at the base of the tail [33].

### 4.2. Treadmill Running

Eight-week-old male DA rats were randomly divided into four groups: Normal rat sedentary group (control), normal rat treadmill running group (treadmill), CIA rat sedentary group (CIA), and CIA rat treadmill running group (CIA + treadmill). Two weeks after induction of CIA in the CIA and CIA + treadmill groups, the treadmill group and the CIA + treadmill group of rats were subjected to forced running using a rodent treadmill machine (TMS8D; MEQUEST, Toyama, Japan) at 12 m/min for 4 weeks for 5 days/week, 30 min/day. The other groups (control and CIA groups) were left in the cage. Rats were housed under a 12 h light–dark cycle and allowed food and water ad libitum. Four weeks after starting the treadmill running protocol in the treadmill and CIA + treadmill groups, all rats were sacrificed.

### 4.3. Histochemical Analyses and Semi-Quantitative Analysis of Cartilage Destruction Severity in RA Model

Four weeks after starting the treadmill running protocol in the treadmill and CIA + treadmill groups, all rats were sacrificed and right hind limbs were excised and fixed in 4% paraformaldehyde (Wako, Osaka, Japan), demineralized in 20% ethylenediaminetetraacetic acid, and embedded in paraffin. Sagittal sections, 6μm in thickness, were prepared from the center of the ankle. The sections were stained with hematoxylin and eosin or safranin O. We histologically evaluated arthritic changes, such as infiltration of inflammatory cells, synovial proliferation, destruction of articular cartilage, and bone erosion, as previously described [34]. To investigate the activity of osteoclasts and osteoblasts in vivo, sections were stained with a TRAP/ALP staining kit (Wako Pure Chemical Industries, Osaka, Japan) following the manufacturer’s procedure.

### 4.4. Immunohistochemical Analyses

For immunohistochemistry of TNF-α and Cx43, paraffin-embedded sections were de-paraffinized in xylene, rehydrated through graded alcohol, and immersed in PBS. Endogenous peroxidase activity was blocked by incubating the sections in 3% H_2_O_2_ in methanol for 5 min. The sections were incubated at 4 °C with rabbit polyclonal anti-TNF-α (ab6671, Abcam, Cambridge, UK) at 1:150 or anti-Cx43 (#3512, Cell Signaling Technology, Danvers, MA, USA) at 1:50 overnight. After extensive washing with PBS, the sections were incubated in Histofine Simple Stain Rat MAX-PO (NICHIREI BIOSCIENCES INC., Tokyo, Japan) for 30 min at room temperature. Immunostaining was detected by DAB staining. Counter staining was performed with Mayer’s hematoxylin. The images were analyzed using ImageJ (National Institutes of Health, Bethesda, MD, USA). We randomly extracted from the area where there was evidence of synovial hyperplasia from each slide at 3 regions. The percentage of immunostaining positive tissue within the imaging area was averaged and evaluated semi-quantitatively.

### 4.5. μ-CT Analysis of the Left Ankle

Rat left ankle joints fixed in 70% ethanol were scanned using a µ-CT system (TOSCANER-32300μFD, TOSHIBA, Tokyo, Japan). The reconstructed data sets were examined using three-dimensional data analysis software (TRI/3-D-BON, RATOC System Engineering Co., Tokyo, Japan). The volumes of interest were defined in the trabecular zone in the talus bones. To analyze the talus, the following trabecular bone parameters in the whole talus were evaluated: BV/TV, Tb.Th, and Tb.Sp. Moreover, to quantify bone loss, indirect parameters of microarchitecture were also assessed, including MSV, which is the mean volume of all the parts of an object that can be unobscured in all the directions from a point inside the object [35]. We investigated eroded bone surface per total bone surface on talus and osteophyte volume per whole talus using TRI 3D-BON software (RATOC System Engineering Co., Tokyo, Japan).

### 4.6. Statistical Analysis

All data are presented as the mean and standard deviation (SD). We analyzed all data using analysis of variance (ANOVA), and performed post-hoc testing using the Tukey–Kramer test. The non-parametric Mann–Whitney *U*-test was used to evaluate the statistical significance of differences in histological scores. In all analyses, we defined *p* < 0.05 as statistically significant.

## 5. Conclusions

To our knowledge, this study is the first to show that treadmill running in CIA rats suppresses the destruction of joints and improves osteoporosis. This mechanism may involve TNF-α and Cx43, which were suppressed in the CIA + treadmill running group compared with the CIA group. Treadmill running may play an important role in the suppression of pro-inflammatory cytokine production. We expect that exercise therapy, including treadmill running, from the early stage of disease is also important in patients with RA.
